# Who wants a slimmer body? The relationship between body weight status, education level and body shape dissatisfaction among young adults in Hong Kong

**DOI:** 10.1186/1471-2458-11-835

**Published:** 2011-10-31

**Authors:** Yee Tak Derek Cheung, Antoinette Marie Lee, Sai Yin Ho, Edmund Tsze Shing Li, Tai Hing Lam, Susan Yun Sun Fan, Paul Siu Fai Yip

**Affiliations:** 1 Centre for Health Policy, Programs and Economics, School of Population Health, The University of Melbourne, Melbourne, Australia; 2 Department of Psychiatry, The University of Hong Kong, Hong Kong SAR, PR China; 3 Department of Community Medicine, The University of Hong Kong, Hong Kong SAR, PR China; 4 School of Biological Sciences, Faculty of Science, The University of Hong Kong SAR, PR China; 5 Family Planning Association of Hong Kong, Hong Kong SAR, PR China; 6 Department of Social Work and Social Administration, The University of Hong Kong, Hong Kong SAR, PR China

## Abstract

**Background:**

Body shape dissatisfaction has been thought to have an indispensable impact on weight control behaviors. We investigated the prevalence of body shape dissatisfaction (BSD) and explored its association with weight status, education level and other determinants among young adults in Hong Kong.

**Methods:**

Information on anthropometry, BSD, and socio-demographics was collected from a random sample of 1205 young adults (611 men and 594 women) aged 18-27 in a community-based household survey. BSD was defined as a discrepancy between current and ideal body shape based on a figure rating scale. Cross-tabulations, homogeneity tests and logistic regression models were applied.

**Results:**

The percentages of underweight men and women were 16.5% and 34.9% respectively, and the corresponding percentages of being overweight or obese were 26.7% and 13.2% for men and women respectively. Three-quarters of young adults had BSD. Among women, 30.9% of those underweight and 75.5% of those with normal weight desired a slimmer body shape. Overweight men and underweight women with lower education level were more likely to have a mismatch between weight status and BSD than those with higher education level. After controlling for other determinants, underweight women were found to have a higher likelihood to maintain their current body shapes than other women. Men were found to be less likely to have a mismatch between weight status and BSD than women.

**Conclusions:**

Overweight and obesity in men and underweight in women were prevalent among Hong Kong young adults. Inappropriate body shape desire might predispose individuals to unhealthy weight loss or gain behaviors. Careful consideration of actual weight status in body shape desire is needed in health promotion and education, especially for underweight and normal weight women and those with a low education level.

## Background

According to the self-discrepancy theory, body shape dissatisfaction (BSD) results from the discrepancies between perceived current body shape and ideal body shape based on self-evaluation standards [1-3]. BSD has been found to be associated with adverse outcomes including eating disorders and depressive symptoms [4-6]. The associated anxiety and distress may lead to irrational weight-loss behaviors for achieving the desired body shape.

Societal norms within unique ethnic and social communities has significant influence on ideal body shape [3,7,8]. The cultural ideal shape differs between men and women, which in turn is heavily influenced by the media [9-11]. For instance, BSD is common among women and is reflected in their constant desire for a slimmer body irrespective of their actual body shape [10-12]. Young women consider their body weight "about right" when their perceived body size is closer to the cultural ideal [10]. A mismatch between BSD and actual weight status exists when underweight people desire to be thinner or maintain the current body shape. This mismatch also occurs when normal weight people do not want to maintain their current body shapes, and overweight or obese people do not desire to have a slimmer body shape. Some reported that East Asian women were more concerned about their facial appearance than body shape and found it easier to attain thinner bodies than Western women do [13], but these findings were not supported by a recent empirical study on Chinese young women [8].

Males, on the other hand, generally prefer a more muscular body shape which is also not necessarily linked to body weight status [14,15]. Body image perceptions among males changes with age [7]. Pre-adolescent boys are generally satisfied with their body but their desire for a bigger build increase as they grow up to adolescence stage. During adulthood, men are evenly split in their desire to gain or lose weight, while underestimation of body shape is still more prevalent among younger men than older men [16]. Compared with females, however, there have been fewer studies on BSD among Asian males. With a growing prevalence of overweight and obesity worldwide [17-19]; as well as massive advertisement by slimming centers, deeper understanding on BSD and its determinants among contemporary Chinese men is needed.

Most previous BSD studies used samples of adolescents and students from colleges [2,10,12,20,21]. These non-probability convenient samples could hardly represent young adults in general. We conducted a community-based survey among Chinese young adults, based on a representative sample, to examine the prevalence of BSD by sex and weight status, and explored the associations of BSD with weight status and socio-demographic factors. We hypothesized that BSD differs by sex, and is not necessarily matched with the actual weight status among young adults. We also used predictive models to explore the multivariate influence of weight status and socio-demographic factors on BSD. Besides desiring a slimmer and bigger body shape, attitude of maintaining the current body shape is also treated as one dependent variable in the multivariate analysis.

## Methods

### Ethics statement

The survey and data analysis was approved by the Research Subcommittee of the Family Planning Association of Hong Kong. This study was supported by the University Research Committee Strategic Research Theme (SRT) of Public Health, The University of Hong Kong.

### Sample

The Youth Sexuality Study is the longest running community-based sexuality survey in Hong Kong. The objective of the study is to assess sexual knowledge and attitude among young adults in the community. The survey was first conducted in 1986 and from then on once every 5 years. The survey data obtained in 2006 was used for data analysis of the current study. Our sample was obtained from the Census and Statistics Department under the Hong Kong SAR government who had a complete sampling frame of all living quarters in Hong Kong. Upon our request for about 10,000 living quarters, that department used a systematic sampling method to draw samples from the sampling frame. For each selected living quarter, we used Kish grid method to randomly select one household member who was aged 18 to 27 as respondent to fill in a self-administered questionnaire at his/her own time and return it before a specific time. Among the 10,001 living quarters being selected and visited, 2,369 had no one at home at the time of visit, were rejected by the first contact person of the living quarter, or had invalid addresses. Of the remaining 7,632 living quarters, 1,356 households contained eligible respondents. Of the eligible respondents, 41 (3%) chose not to participate, 78 (5.8%) dropped out half-way, and 32 (2.4%) did not return the questionnaire, leaving 1,205 young adults (88.9%) who successfully completed the questionnaire.

### Measures

#### Demographics

Basic demographic information including age, education level and employment status were collected. Education level was classified as junior secondary (Form 3 or below, equivalent to year 9 or below), senior secondary (Forms 4 to 5, equivalent to years 10 and 11), matriculation or high diploma or associate degree, and undergraduate degree or above. Employment status was classified as full-time students, full-time workers, unemployed and housewives. As the numbers of unemployed and housewives were only 56 (4.7%) and 20 (1.7%) respectively, we aggregated the two groups into one called "unemployed" in the multivariate analysis.

### Body mass index (BMI) and weight status

Body mass index (BMI), calculated as weight (in kg) divided by height squared (m^2^), was used to define weight status according to the WHO adult BMI standard for Asians [22] (Table 1). To facilitate the large sample collection and save cost, self-reported height and weight were used to compile the BMI and categorize weight status. Since the use of self-reported anthropometry data has been criticized for underestimating the prevalence of obesity when compared with measured data [23,24], we compared our results with another study, in which the height and weight were measured with valid and calibrated weighing scale and tape measures [25]. That study reported that the prevalence rates for age groups of 15-24 and 25-34 were 25.1% and 13.8% for underweight, 8.7% and 11.6% for overweight, and 5.9% and 13.4% for obesity, respectively. These figures were similar to ours and hence supported the utilization of our self-reported data.

**Table 1:** Demographics and weight-related characteristics by sex

	Men	Women	All	Test for sex difference
	(n = 611)	(n = 594)	(n = 1,205)	
Mean age (years)	21.97	22.04	22.0	
Marital status (%)				p < 0.01
Single	91.0	84.6	87.8	
Married or cohabited	7.7	12.8	10.2	
Divorced or separated	0.5	2.1	1.3	
Widowed	0.9	0.5	0.7	
Employment status (%)				p < 0.01
Full-time student	42.1	38.3	40.2	
Full-time workers	53.6	53.2	53.4	
Unemployed	4.3	5.1	4.7	
Housewives	0.0	3.4	1.7	
Education attainment (%)				p < 0.01
Junior secondary or below	11.3	4.7	8.3	
Senior secondary	34.7	34.3	34.5	
Matriculation or Vocational training	29.3	34.0	31.6	
Undergraduate degree or above	24.2	27.0	25.6	
Mean BMI (95% confidence interval)	21.63	19.99	20.81	p < 0.01
	(21.30, 21.96)	(19.72, 20.25)	(20.59, 21.02)	
Weight status (%)				p < 0.01
Underweight	16.5	34.9	25.7	
Normal weight	56.7	51.9	54.3	
Overweight	11.3	7.3	9.3	
Obese	15.4	5.9	10.7	

	Mean score and 95% confidence interval			

Current body shape	4.33	3.68	4.01	p < 0.01
	(4.21, 4.45)	(3.58, 3.78)	(3.92, 4.09)	
Ideal body shape	4.38	2.93	3.65	p < 0.01
	(4.31, 4.45)	(2.87, 2.99)	(3.59, 3.72)	
Body shape dissatisfaction (Current-Ideal)	-0.05	0.75	0.35	p < 0.01
	(-0.17, 0.07)	(0.66, 0.84)	(0.27, 0.43)	
Absolute value of body shape dissatisfaction	1.11	1.01	1.07	p = 0.08
				
	(1.05, 1.20)	(0.94, 1.09)	(1.01, 1.12)	

Chi-square tests were used for categorical variables, and independent samples t-tests were used for continuous variables.

### Body shape dissatisfaction

**Figure 1:**
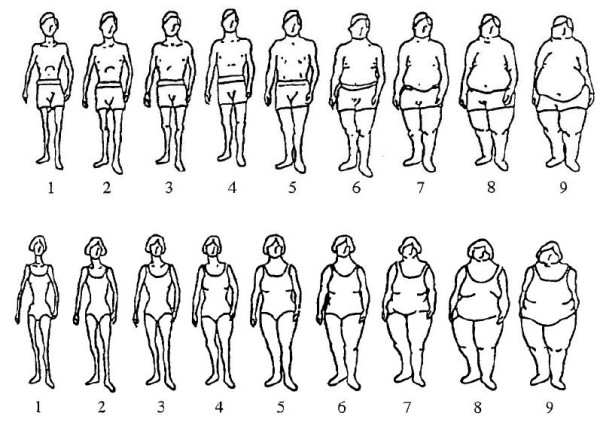
**The figure rating scale of 9 sex-specific body silhouettes**.

BSD was measured with a rating scale from Stunkard visual figures which consists of nine gender-specific body silhouettes ranging from very thin (value = 1) to very big (value = 9) (Figure 1). Validity in this figure rating scale was affirmed by a previous research study [26], and was found to have good test-retest reliability [27]. Respondents were asked to choose the figure that best represented their "current shape" and then their "ideal shape". The absolute value of BSD was derived from the discrepancies between the chosen current and ideal figures, regardless of direction of thinness or fatness preference. Respondents were then classified into three groups: (1) desired to maintain the current body shape (current = ideal), (2) desired to have a slimmer body shape (current > ideal), and (3) desired to have a bigger body shape (ideal > current).

### Statistical analysis

Descriptive statistics were used to illustrate the distribution of BMI, weight status, body shape preferences and BSD. To examine sex differences, chi-square tests were conducted to test the differences for categorical variables, and independent samples t-tests were used for continuous variables. Furthermore, the relations of BSD with education level and employment status were examined for each weight status with chi-square tests.

Three multiple logistic regressions were conducted to calculate odds ratios of desire to maintain the current body shape, desire to have a slimmer body shape, and desire to have a bigger body shape for weight statuses based on BMI, age, education level and employment status for each sex. For each regression model, other respondents not in the response category were treated as the reference category. Adjusted odds ratios were obtained to estimate the predictive power of each variable. Infinite or zero odds ratios, with very high p-values, were obtained for overweight and obese in the women's regression models because all overweight and obese women respondents desired a slimmer body shape. In the final model, therefore, we aggregated women of normal weight, overweight and obese into one category for a more robust estimation. The Statistical Package for the Social Science (SPSS version 16.0) was used for all the analyses.

## Results

### Distribution of actual weight status and BSD

Overall, 25.7% of the young adults were classified as underweight, 9.3% were overweight, and 10.7% were obese. Underweight was more common in women while overweight or obesity were more common in men. More men were obese (15.4%) than were overweight (11.3%). Significant sex differences were observed for BMI, current body shape and ideal body shape. BSD was found to be significantly greater for women than men (*t *= -11.065, *p *> 0.01). When the absolute value of BSD was compared, the sex difference was no longer statistically significant (*t *= 1.764, *p *= 0.078).

**Figure 2:**
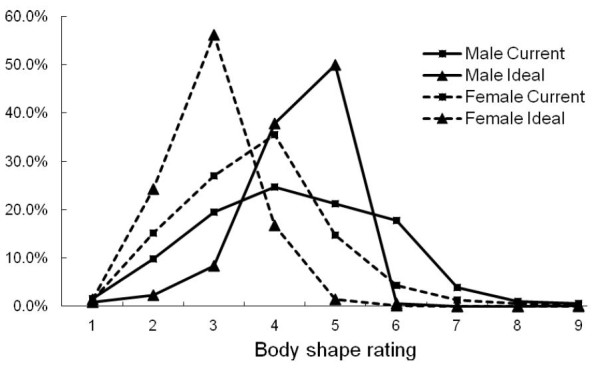
**Distribution of current and ideal body shape rating by sex**.

For women, current body shape ratings of 3 and 4 were the most common (Figure 2). For men, the ratings were more evenly distributed from 3 to 6. Most women had an ideal body shape rating of 2, while most men desired higher ratings of 4 or 5. No subjects chose ratings of 6 or above as their ideal body shape.

More women than men desired a body shape slimmer than their current one (63.3% vs. 38.4%, *t *= -9.187, *p *< 0.01), whereas more men than women desired a bigger body shape (38.4% vs. 11.3%, *t *= 11.44, *p *< 0.01) (Table 2). Similar proportions of men (23.2%) and women (25.5%) wished to maintain their current body shape (*t *= -.592, *p *= 0.55). However, the distribution for ideal body shape was more symmetrical for men but was right-skewed for women. (Figure 2)

**Table 2:** Rates of body dissatisfaction by weight status and sex

		Desired to maintain current body shape (%)		Desired to have a slimmer body shape (%)		Desired to have a bigger body shape (%)	
Men	Underweight (n = 90)	21	23.3	11	12.2	58	64.4
	Normal weight (n = 309)	85	27.5	84	27.2	140	45.3
	Overweight (n = 63)	15	23.8	42	66.7	6	9.5
	Obese (n = 85)	6	7.1	73	85.9	6	7.1
	Subtotal (n = 547)	127	23.2	210	38.4	210	38.4
	Chi-square test	$\chi_{3}^{2}=15.653$, p < 0.01		$\chi_{3}^{2}=144.814$, p < 0.01		$\chi_{3}^{2}=89.554$, p < 0.01	

Women	Underweight (n = 191)	77	40.3	59	30.9	55	28.8
	Normal weight (n = 286)	63	22.0	216	75.5	7	2.4
	Overweight (n = 40)	0	0.0	40	100.0	0	0.0
	Obese (n = 33)	0	0.0	33	100.0	0	0.0
	Subtotal (n = 550)	140	25.5	348	63.3	62	11.3
	Chi-square test	$\chi_{3}^{2}=48.922$, p < 0.01		$\chi_{3}^{2}=147.037$, p < 0.01		$\chi_{3}^{2}=90.181$, p < 0.01	

All percentages were row percentages within a certain group of gender and weight status

#108 respondents (9%) were omitted from the analysis as they did not provide sufficient information for compilation of either BMI or BSD

### Mismatch between weight status and body shape dissatisfaction

Among men who had BSD, about half of them desired to have a slimmer body and half of them desired to have a bigger one (Table 2). We observed that (i) 23.3% of those underweight desired to maintain the current body shape, (ii) 12.2% of underweight and 27.2% of normal weight desired a slimmer body, and (iii) 45.3% of men with normal weight desired a bigger body shape. For women, (i) 40.3% of those underweight expressed no BSD and 30.9% desired an even slimmer body shape despite being underweight. In other words, 71.2% of those underweight did not desire a bigger body shape, and (ii) 75.5% of those normal weight desired a slimmer body shape.

### Determinants of desiring slimmer body shape

**Figure 3:**
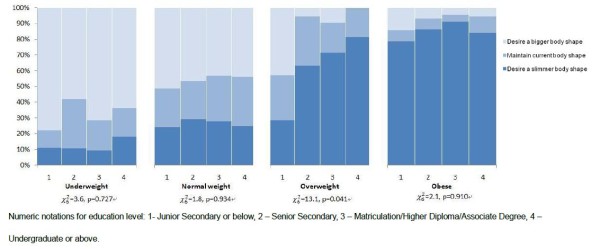
**Distribution of body shape dissatisfaction by education level and BMI weight status among male respondents**.

**Figure 4:**
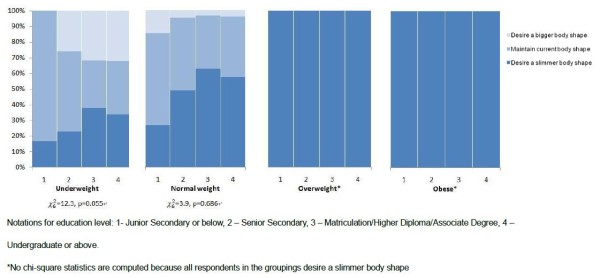
**Distribution of body shape dissatisfaction by education level and BMI weight status among female respondents**.

Figures 3 and 4 depict the distribution of BSD by education level for men and women respectively. Men who were overweight and with lower education level were less likely to desire a slimmer body shape than those overweight men with higher education level (Figure 3). Underweight women with lower education level were more likely to be, with marginally statistical significance, satisfied with their current body shape (Figure 4). Most underweight women with junior secondary education level or below were satisfied with their body (83.3%), compared with only 34% among their counterparts with educational attainment at undergraduate level or above. On the other hand, underweight women who were full-time students were more likely to desire a bigger body shape (37.7% vs 24.3%), and less likely to desire to maintain their current body shape than full-time working respondents (28.6% vs 45.6%) ($\chi_{4}^{2}=9.2$, p = 0.057). Such difference by employment status was not apparent among female respondents who were normal weight, overweight, and obese, nor among male respondents of any weight category.

For men, after controlling for socio-demographic variables in the multiple regression models, underweight significantly predicted the desire of a bigger body shape, while overweight and obese significantly predicted the desire of a slimmer body shape (Table 3). Obese men had 5-fold decreased odds than normal weight men to desire to maintain the current body shape, but there were no significantly increased or decreased odds of maintaining the current body shape for underweight and overweight men. For women, the underweight group was less likely to desire a slimmer body than women in other weight status categories. However, underweight status had 3-fold increased odds of maintaining the current body shape than women with other weight status (AOR = 3.17, p-value < 0.01). Interactions for weight status, education level, and employment status were tested but no significant findings were obtained.

**Table 3:** Adjusted odds ratios for maintaining the current body shape and BSD by demographic and anthropometric characteristics derived from multiple logistic regressions

Male						
**Response category**	**Desired to maintain current body shape**		**Desired to have a** **slimmer body shape**		**Desired to have a** **bigger body shape**	
	**AORs**	**95% CI**	**AORs**	**95% CI**	**AORs**	**95% CI**

Age	1.04	(0.95,1.14)	0.92	(0.84,1.01)	1.04	(0.96,1.14)
Weight status						
Underweight	0.78	(0.45,1.38)	0.36	(0.18,0.72)	2.34	(1.42,3.87)
Normal weight	1.00		1.00		1.00	
Overweight	0.83	(0.44,1.58)	5.95	(3.26,10.86)	0.10	(0.04,0.27)
Obese	0.20	(0.09,0.48)	18.16	(9.17,35.98)	0.09	(0.04,0.21)
Employment status						
Full-time workers	0.72	(0.41,1.30)	1.19	(0.67,2.14)	1.16	(0.68,1.99)
Unemployed	1.36	(0.46,4.09)	0.79	(0.22,2.87)	0.86	(0.29,2.6)
Full-time students	1.00		1.00		1.00	
Educational level						
Junior secondary or below	0.94	(0.42,2.13)	0.54	(0.24,1.22)	1.77	(0.84,3.76)
Senior Secondary	1.15	(0.65,2.05)	0.84	(0.48,1.51)	1.03	(0.60,1.80)
Matriculation/AD/HD	0.97	(0.55,1.71)	0.88	(0.5,1.56)	1.14	(0.67,1.96)
Undergraduate degree or above	1.00		1.00		1.00	

Female						

Response category	Desired to maintain current shape		Desired a slimmer body shape		Desired a bigger body shape	
	AORs	95% CI	AORs	95% CI	AORs	95% CI

Age	1.03	(0.95,1.14)	0.96	(0.88,1.06)	1.00	(0.87,1.15)
Weight status						
Underweight	3.21	(2.15,4.83)	0.11	(0.08,0.17)	20.61	(9.12,46.62)
Normal weight/Overweight/Obese	1.00		1.00		1.00	
Employment status						
Full-time workers	1.20	(0.67,2.15)	1.15	(0.65,2.05)	0.58	(0.26,1.31)
Unemployed	2.32	(0.93,5.81)	0.72	(0.28,1.89)	0.19	(0.03,1.69)
Full-time students	1.00		1.00		1.00	
Educational level						
Junior secondary or below	1.88	(0.72,4.99)	0.61	(0.23,1.69)	0.49	(0.06,4.53)
Senior Secondary	1.08	(0.63,1.88)	0.95	(0.55,1.66)	0.89	(0.40,2.02)
Matriculation/AD/HD	0.85	(0.49,1.48)	1.25	(0.73,2.14)	0.82	(0.38,1.79)
Undergraduate degree or above	1.00		1.00		1.00	

## Discussion

To the best of our knowledge, this is the first study which utilizes a population-based sample to examine the pattern of weight status and BSD among young adults in a Chinese society. Previous studies on body image dissatisfaction among Asian women suggested that thinness and fragility were crucial components of feminine beauty throughout traditional Chinese society [28]. Our current study aimed to understand the body shape dissatisfaction among men and women in the contemporary Hong Kong society. We found that the distribution of ideal body shape for women generally shifted towards slimmer body shape, compared with the curve of perceived current body shape. This means that women generally perceive their current body shape bigger than what they desire. On the contrary, both curves of perceived current and ideal body shapes for men were more alike and peaked at body shape rating 5. Men expressed the same extent of body shape dissatisfaction as women did, but there were greater heterogeneity in their desire of being slimmer or bigger, which is quite consistent with western men [7,10]. These findings are coherent to the fact that body shape dissatisfaction, for both men and women, is inevitably taking place in Hong Kong, where is under the influence of traditional Chinese values and westernization [28,29].

### Mismatch between actual weight status and body shape satisfaction

There are two competing views on the relationship between actual physical status (e.g. BMI) and subjective body image evaluations. Some studies have found that BMI is an important determinant of body satisfaction [10,21,30], while others have argued that objective indices such as BMI are less important than psychological constructs such as self-esteem in affecting body dissatisfaction [2,8,10]. Our findings supported the association, though we also identified the existence of "mismatch" between actual weight status and body shape dissatisfaction [31,32]. The majority of the female respondents with normal BMI weight status still desired for a slimmer body. Those women who had achieved healthy body mass were not necessarily more satisfied with their body shapes than those who were underweight, while underweight women were even more likely to consider their body weight as "about right" and expressed the smallest disagreement between current and desired body shape [10,12]. This mismatch is coherent to the fact that desire for ideal body shape is highly biased by sociocultural norms, environmental neighborhood affluence and media impact for women [7,9,20,33]. On the other hand, men also had the mismatch but to a less extent than women. Similar to women, men with normal weight did not have higher intention than underweight and overweight men to maintain their current body shapes. Although nowadays having BSD is common in developed and affluent societies, the actual weight status should be taken into account in making any choice of actions to acquire the desired body shape. The emphasis on subjective judgment rather than objective indicators on current body shape desire might lead to unnecessary body weight reduction strategies and adverse psychological distress among the general public. This misperception of BMI and current body shape could be a major problem and further studies on this are warranted. To avoid such public health crisis arising from current and ideal body shape misconception, public health education aiming to raise awareness on actual weight status in desiring body shape is necessary.

### Education level and body shape dissatisfaction

One previous study has found that poor body satisfaction was more prevalent among women with higher education attainment than women with lower education attainment [34]. On the other hand, the impact of obesity on women's general health was greater among those with a lower education level [35]. Our study attempted to explore the role of education attainment and employment status on the mismatch between weight status and body dissatisfaction, with an extension to male sample. Our findings showed that overweight men with lower education level had less desire for a slimmer body shape than their counterparts with higher education level. For women, the desire to stay in the current body shape was more common among the underweight with lower education level, compared to their counterparts with higher education level. These findings supported that the role of education level on BSD is not only influential to women but also to men. Both overweight men and underweight women with low education level tended to neglect actual weight status in pursuit of an ideal body shape. The possible explanation is that these people with lower education level lack awareness about their actual weight status due to insufficient knowledge of determining fatness and thinness. Both overweight and underweight population with a low education level should be targeted in educational programs regarding body weight control.

### Underweight problem among young adults

Since obesity and underestimation of weight status have been very much emphasized within the community and public health constituencies globally [16,36], relatively less attention has been paid to the underweight population and its health consequences in the territory. Our study showed that one-fourth of the young adults were underweight (BMI<18.5), compared with 9.3% and 10.7% who were overweight and obese respectively. Our findings are coherent with the recent territory-wide based Population Health Survey which found similar underweight prevalence in the territory [25]. In that study, overall 10.3% of the community were underweight based on BMI, and 28.8% were overweight or obese. However, among the age group of 15-24, the corresponding figures were 25.1% and 13.8%, respectively. These figures supported that underweight is as prevalent as overweight and obese among young adults in the community. Media induced perception on the myth of a slimmer self-body image through TV-ads, fashion magazines, beauty contests, and reinforcement of culturally idealizing slimness by diet and fashion industries could also be the possible factors underlying the desire of the slim ideal image and weight-loss behaviors [37]. Indeed, underweight among young adults is associated with adverse health outcomes such as maladaptive weight loss behaviors, disordered eating and other mental health consequences [8,38]. Those underweight who desired to have a slimmer body shape could be a high-risk group for these adverse outcomes.

### Limitations of the study

Although our study yielded a representative youth sample, the small sample size may limit the reliability of our statistical estimate. For instance, only 8.3% of the respondents reported an education level below junior secondary school. Hence, our conclusion about the impact of low education level on BSD can only be drawn on this group with a small sample size. We suggest that a larger sample size and proportion of people having a low education level would be needed for more in-depth analysis to understand how education level is related to BSD.

Methodological issues about the BMI cut-off and the use of figure rating scale of this study should also be acknowledged. BMI cut-off for obesity is directly related to various health risks and risk factors of morbidity and mortality [39], however, there are fewer consensus on cut-offs for underweight in adults [40]. Using the cutoff value of 18.5, the WHO-defined thinness grade I (mild thinness), as the cut-off for underweight, 25.7% of our subjects were underweight. The WHO expert committee, however, has pointed out that though the single cut-off point of 18.5 for specified mild deficiency was a reasonable value to define underweight, it has little experimental support [41]. Especially for men aged 18 to 25, their average BMI were lower than that of men aged 26 to 40. In a recent study on definition of thinness among young adolescents, the universal cut-off BMI for underweight children at age 18 was more suitably set at 17 [40]. Using the value of 17 as the cut-off, only 6.3% of men and 10.4% of women of our respondents were underweight. Such substantial difference owing to the use of different cut-offs of underweight would result in large discrepancies in assessing prevalence of underweight and its associated health risk. Thus, further work need to be conducted to develop an empirically based cut-off for underweight among young adults in Asia.

There are two main measurement tools to assess body image satisfaction, (i) assessment of satisfaction with specific body parts [2,8,21,42,43].; and (ii) Figure rating scale (FRS) [10,44]. Specifically on body shape dissatisfaction, Neighbors & Sobal (2007) applied a figure rating scale (FRS) which requires respondents to choose their actual perceived and ideal body shape from a series of body silhouettes of increasing body size and shape, with body shape dissatisfaction being operationalized as the discrepancies between actual and ideal body shapes shown on the questionnaire [26]. Measurement tool (ii), which is the tool being applied in our study, is more relevant to our research questions to assess overall body shape than (i). However, it should be noted that the body silhouettes were based on Western norms and its suitability for Asian Chinese is yet tested. Since a standard weight status according to BMI for Asians is already in use, developing an ethnically specific FRS would be particularly worthwhile.

## Conclusions

Overall, a substantial proportion of underweight is found among the young adults in Hong Kong which has often been overlooked. We found that women had a higher prevalence of underweight, but lower prevalence of overweight or obese compared to men. Regarding body dissatisfaction, similar proportions of men desired bigger or smaller body shapes, while most women desired a slimmer body shape. We also observed a mismatch between weight status and body dissatisfaction, such that the desire for an ideal body shape which may precipitate unhealthy weight gain or loss. In addition, we found that overweight men and underweight women with lower education level were more likely to have such mismatch than counterparts with higher education level. These findings signal the call for more effective interventions and educations targeted towards those who potentially desire inappropriate body shape.

## Competing interests

The authors declare that they have no competing interests

## Authors' contributions

YTDC contributed to literature review, study design, performed statistical analyses and drafted the manuscript; AML, SYH, ETSL, THL and PSFY made revisions to the manuscript; AML, SYH, THL, SF and PSFY contributed to questionnaire design and coordination of the household survey; PSFY oversaw the development of the research study. All authors read and approved the final manuscript.

## Pre-publication history

The pre-publication history for this paper can be accessed here:

http://www.biomedcentral.com/1471-2458/11/835/prepub
